# Mask use in community settings in the context of COVID-19: A systematic review of ecological data

**DOI:** 10.1016/j.eclinm.2021.101024

**Published:** 2021-07-19

**Authors:** Nathan Ford, Haley K. Holmer, Roger Chou, Paul J. Villeneuve, April Baller, Maria Van Kerkhove, Benedetta Allegranzi

**Affiliations:** a Guidelines Review Committee, World Health Organization, Geneva, Switzerland; b Center to Improve Veteran Involvement in Care (CIVIC), VA Portland Health Care System, Portland, OR, United States; c Pacific Northwest Evidence-based Practice Center and Oregon Health & Science University, Portland, OR, United States; d School of Mathematics and Statistics and Department of Neuroscience, Faculty of Science Carleton University, Ottawa, Canada; e Health Emergencies Programme, World Health Organization, Geneva, Switzerland; f Infection Prevention and Control Technical and Clinical Hub, Department of Integrated Health Services, World Health Organization, Geneva, Switzerland

## Abstract

**Background:**

The wearing of medical and non-medical masks by the general public in community settings is one intervention that is important for the reduction of SARS-CoV-2 transmission, and has been the subject of considerable research, policy, advocacy and debate. Several observational studies have used ecological (population-level) data to assess the effect of masks on transmission, hospitalization, and mortality at the region or community level.

**Methods:**

We undertook this systematic review to summarize the study designs, outcomes, and key quality indicators of using ecological data to evaluate the association between mask wearing and COVID-19 outcomes. We searched the World Health Organization (WHO) COVID-19 global literature database up to 5 March 2021 for studies reporting the impact of mask use in community settings on outcomes related to SARS-CoV-2 transmission using ecological data.

**Findings:**

Twenty one articles were identified that analysed ecological data to assess the protective effect of policies mandating community mask wearing. All studies reported SARS-CoV-2 benefits in terms of reductions in either the incidence, hospitalization, or mortality, or a combination of these outcomes. Few studies assessed compliance to mask wearing policies or controlled for the possible influence of other preventive measures such as hand hygiene and physical distancing, and information about compliance to these policies was lacking.

**Interpretation:**

Ecological studies have been cited as evidence to advocate for the adoption of universal masking policies. The studies summarized by this review suggest that community mask policies may reduce the population-level burden of SARS-CoV-2. Methodological limitations, in particular controlling for the actual practice of mask wearing and other preventive policies make it difficult to determine causality. There are several important limitations to consider for improving the validity of ecological data.

**Research in context**

**Evidence before this study**

The wearing of medical and non-medical masks by the general public in community settings is an important intervention to reduce SARS-CoV-2 transmission. Much of the evidence supporting the protective benefit of mask wearing is derived from studies of other respiratory pathogens in health care settings, with only a limited number of studies reporting direct outcomes on the protective wearing of face masks by the community to prevent SARS-CoV-2 transmission. A larger number of studies have been published based on ecological data, but such studies are subject to important methodological limitations.

**Added value of this study**

This is the first review to systematically assess the available ecological (country or regional) data evaluating the association between mask wearing and COVID-19 outcomes and assess the quality of this body if evidence. All studies identified by this review reported a protective benefit in terms of either reduced incidence, mortality, hospitalization, or a combination of these outcomes. However, few studies provided any information about where masks were worn and by whom, type of mask (medical or non-medical), rate of mask wearing and level of compliance, and studies were limited in their ability to control for other infection control measures and confounders.

**Implications of all the available evidence**

The results of ecological studies in this review provide supportive evidence about the protective effect of mask wearing in community settings, and this body of evidence suggests that mask policies may reduce the population-level burden of SARS-CoV-2 in non-vaccinated populations. Future research should consider approaches to improving the validity of ecological data to inform policy and practice, in particular to be able to draw conclusions about the relative effectiveness of different nonpharmaceutical public health measures.

Alt-text: Unlabelled box

## 1 Introduction

The wearing of medical and non-medical masks by the general public in community settings for prevention of respiratory virus transmission has been the subject of considerable debate, research, policy and advocacy. Since January 2020, WHO has recommended the use of masks as part of a comprehensive control strategy to suppress transmission of SARS-CoV-2 for health workers and the general public and this advice has been updated regularly [1].

At the start of the pandemic, much of the evidence supporting the protective benefit of mask wearing was derived from studies of other respiratory pathogens, in particular seasonal influenza, severe acute respiratory syndrome (SARS) and Middle East respiratory syndrome (MERs) [2], [3], [4]. A limited number of studies have reported outcomes on the wearing of face masks by the community to prevent SARS-CoV-2 transmission, including one randomized trial [5] and several comparative and non-comparative observational studies [6], [7], [8].

A larger number of observational studies have used ecological (population-level) data to assess the effect of masks on virus transmission at the region or group level [9]. Ecological studies are often conducted using routinely collected data, such as country or regional data and can provide some evidence on the effectiveness of policies on disease progression and other outcomes.

The findings of ecological studies are often used to make strong causal inferences about individual and contextual effects. However, data aggregation can lead to cross-level bias – that average characteristics of the group apply to individuals (also known as the ecological fallacy) [10]. Due to known limitations, ecological studies have generally been excluded from systematic reviews of studies assessing community mask use [2,8,11]. However, ecological studies of mask policies may provide useful information, regardless of potential limitations, in an emergency event such as COVID-19, when there is low certainty evidence and end users seek to make rapid decisions based on all available evidence.

We undertook this systematic review to summarize the study designs, outcomes, and key quality indicators of using ecological data to evaluate the association between mask wearing and COVID-19 outcomes. To our knowledge, this is the first systematic review of these studies.

## 2 Methods

This study has been designed and reported according to the Preferred Reporting Items for Systematic Reviews and Meta-Analysis (PRISMA) statement [12]. The protocol for this study, including search strategy, is available in the Supplementary appendix. Using a highly sensitive search strategy developed by an information specialist, we searched the WHO COVID-19 global literature database for studies reporting the impact of mask use in community settings on outcomes related to SARS-CoV-2 transmission using ecological data. The WHO COVID-19 Research Database (https://search.bvsalud.org/global-literature-on-novel-coronavirus-2019-ncov/) was created in January 2020 in response to the need for a centralized repository of citations relevant to the current pandemic. The database is a compilation of over 40 searches conducted in resources such as PubMed, Embase, CINAHL, GIM. A full list of resources is listed here (https://www.who.int/docs/default-source/coronaviruse/who-covid-19-database/who-covid-19_sources_searchstrategy_20210105.pdf?sfvrsn=480292c0_9). Outcomes of interest included incidence of SARS-CoV-2, disease severity, and mortality. Studies that evaluate the effects of a masking policy on the population and do not assess both exposure and health outcomes at the individual level are considered ecological studies for this review.

Searches were conducted by a single reviewer (NF) and verified by a second review (HH). Reference lists of published reviews were also screened. From a broad screen of all articles published on the use of masks to prevent SARS-CoV-2 transmission in community settings, we searched for studies that reported on the association between mask wearing and incidence, disease severity, and mortality at the ecological (population or aggregate) level; any aggregate level was included (e.g. state, region or country). The search was conducted from database inception (05 January 2020) to 05 March 2021. No language restrictions were applied. Pre-prints were excluded from review.

Ecological measures can be classified into three types: aggregate (population-level) measures of health outcomes, environmental measures (i.e., physical characteristics of the place in which members of each group live or work) and global measures (attributes of groups or places for which there is no distinct analogue at the individual level, such as population density, level of social disorganization, or the existence of a specific law) [14]. For this review, all studies are considered as aggregate studies.

Information on study characteristics, outcomes, and indicators of study quality were extracted and summarized. All extractions were sent to the original study authors for verification. There is no agreed checklist for assessing the methodological quality of ecological studies. Several papers have outlined issues with study designs commonly used to assess ecological data [15], common sources of bias [14,16,17], and reporting issues [18]. Considering these issues, we assessed risk of bias using an adapted version of the Newcastle Ottawa scale in order to evaluate eight key domains for evaluating ecological studies: representativeness of exposure group, ascertainment of exposure (including measurement of compliance with mask wearing), population exposed, comparability of groups, adjustment for confounders (including policy-level and/or community-level factors), outcome assessment, appropriateness of time lag between mask intervention and outcome assessment, and statistical methodology.

### 2.1 Role of the funding source

There was no funding source for this study. The corresponding author had full access to all the data in the study and had final responsibility for the decision to submit for publication.

## 3 Results

### 3.1 Study characteristics

**Fig. 1:**
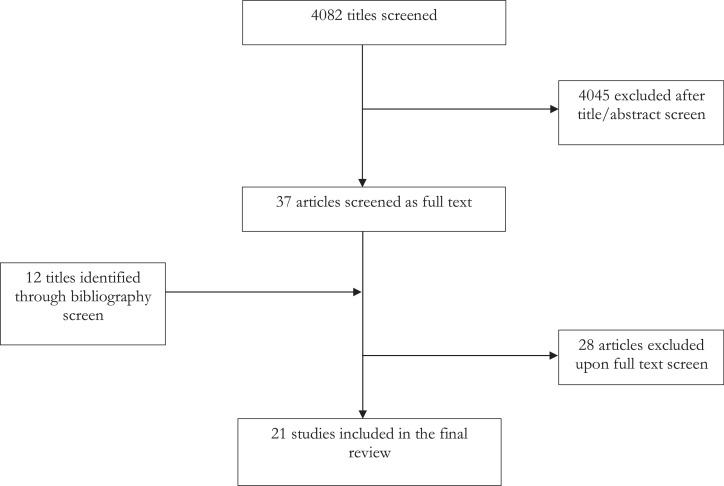
Study selection process.

From an initial screen of 4082 citations, we identified 37 published articles reporting outcomes on the use of masks for the prevention of COVID-19 in community settings. From this set of studies, 21 articles were identified that analysed ecological data to assess the protective effect of masks (Fig. 1) [9,17,[19], [20], [21], [22], [23], [24], [25], [26], [27], [28], [29], [30], [31], [32], [33], [34], [35], [36]]. All studies evaluated the effect of mask policies at the population level; only one study also used individual-level data to assess associations between self-reported mask-wearing and SARS-CoV-2 incidence, assessed through a cross-sectional survey [29]. Five studies compared different countries [21,23,25,28,36], six compared different regions within a single country [20,22,26,[30], [31], [32]], eight undertook a before-after comparison [17,24,25,27,30,[34], [35], [36], [37]], and one compared health workers and the general population [19]. Eleven studies compared the incidence of SARS-CoV-2 across different US States; four studies compared SARS-CoV-2 incidence over time within single US States; one study compared regions in Germany, and the rest compared different countries.

Study characteristics are summarized in Table 1.

**Table 1:** Study Characteristics

Study	Setting	Levels of measurement (Individual, group, region)	Intervention	Comparison	Outcomes	Findings
Mitze et al. [9]	Jena, Germany	Region	Mask mandates: face masks in public transports and shops	Synthetic control	Cumulative incidence	SARS-CoV-2 infections reduced 15–75% over 20 days after mandatory introduction
Lan et al. [19]	Massachusetts, USA	Group (HCW vs state-wide population)	Universal masking policy among HCWs (single healthcare system) and the general public. Daily incidence trends among HCWs	General state population	7-day Temporal incidence trends	Healthcare system's epidemic slope became negative (β −0.68, 95% CI: −1.06 to −0.31), while Massachusetts’ slope remained positive (β: 0.99, 0.94 to 1.05)
Chernozhukov et al. [20]	USA (35 states)	Region	Mask mandates for employees in public businesses	State level data on cases	Cumulative incidence and mortality	Mask mandate could have reduced growth rate of cases and of deaths by approximately 10% in late April, leading to reductions of 21% (90% CI: 9, 32) and 34% (90% CI: 19, 47) in cumulative cases and deaths, respectively, by end of May
Chiang et al. [21]	China (Taiwan) & Singapore	Region	Mask recommendation	National epidemiological data	Cumulative incidence	China (Taiwan) (early) 1.46/100,000 vs Singapore (late) 19.07/100,000
Cheng et al. [22]	Hong Kong	Region	Workplace (mask-on setting)	Non-workplace recreational settings (mask-off setting) Data from other non mask-wearing countries	Cumulative incidence	11 COVID-19 clusters of 133 persons in recreational ‘mask-off’ settings vs 3 clusters of 11 persons in workplace ‘mask-on’ settings
Bo et al. [23]	190 countries	Region	Countries with public mask mandates	Countries that had not (yet) implemented mask mandates	Reproduction number (Rt) (7-day moving average)	Mask mandates associated with −15.4% (95% CI −21.79% to −7.93%) change in Rt
Lyu and Wehby [17]	USA (15 states + Washington DC)	Region	Statewide mask mandates	Pre-post mandate comparison	Daily growth rate	Estimated 230,000–450,000 cases averted
Gallaway et al. [24]	Arizona, USA	Region	Mandatory face masks for community members (via county and city mandates). Enforcement implemented by counties and cities impacted about 85% of the Arizona population	Pre-post mandate comparison	Daily incidence; 7-day moving average	7-day moving average of daily cases decreased 75% from July 13 (3506) to August 7 (867)
Leffler et al. [25]	196 countries	Region	Country-wide mask mandates or cultural norms	Pre-post mandate comparison	Per capita mortality	Weekly increase in per capita COVID-19 mortality: 16.2% (mask mandates) vs 61.9% (no mandates)
Van Dyke et al. [26]	Kansas, USA: 105 counties	Region	Counties with mask mandates	Counties without mask mandates	Daily incidence; 7-day moving average	6% decrease in incidence with mask mandate (mean decrease 0.08 cases/100,000 per day; 95% CI −0.14 to −0.03); 100% increase with no mandate (mean increase 0.11 cases/100,000 per day, 95% CI 0.01 to 0.21)
Kanu et al. [27]	Delaware, USA	Region	Statewide mask mandates	Pre-post mandate comparison	Incidence, hospitalization and mortality	Mask mandates and other measures contributed to reductions in incidence (82%), hospitalizations (88%), and mortality (100%)
Zhang et al. [28]	China, Italy, USA	Region	Countries with mask mandates	Pre-post mandate comparison	Incidence	Wearing of face masks in public is the most effective means to prevent transmission; reduced infections by over 75,000 in Italy and over 66,000 in NYC
Zhang and Warner [33]	USA (50 states)	Region	Statewide mask mandates	Pre-post mandate comparison	Incidence; 7-day rolling average	Mask mandates had a larger effect on flattening the curve than shutdowns based on std coefficient daily infection growth rates. COVID-19 daily average infection growth rate 2.74% with mask mandate vs. 14.35% during shutdowns after 3 weeks
Rader et al. [29]	USA	Region	Self-reported mask wearing; states with mask mandates	Likelihood of wearing mask; pre-post mandate comparison	Community transmission control (Rt <1)	Self-reported mask-wearing associated with a higher probability of transmission control (OR 3.53; 95% CI 2.03 to 6.43)
Li et al. [30]	New York and Massachusetts, USA	Region	Mask mandate in New York	Pre-post mandate comparison; Massachusetts as comparison state	Average daily incidence and mortality	Average daily number of confirmed cases in New York decreased by 2356 (95% CI, 451–4261) after the Executive Order took effect (trend change of 341 cases/day (95% CI 187 to 496)) Average daily number of deaths decreased by 307 (95% CI, 205–410) deaths and the trend decreased by 52 (95% CI, 44–60) deaths per day For the daily cases, the difference in the level change is −2686 (−4961, −412) and the difference in the trend change is −223 (−366, −80). For the daily number of deaths, the difference in the level change is −351(−502, −201), and the difference is −45 (−55, −36)
Rebeiro et al. [31]	USA (50 States and District of Columbia)	Region	Statewide mask mandates	Pre-post mandate comparison	Incidence	Protective effect comparing early to never adopter states: adjusted ratio of incidence rate ratios (aIRRR)= 0.15, 95% CI 0.09–0.23 Lower post- vs. pre-mask case rate slopes, with −1.08% per 100,000 per day (95% CI −1.48%, −0.67%) among early-adopter and −0.37% per 100,000 per day (95% CI −0.86%, 0.10%) among late-adopter versus never-adopter states
Krishnamachari et al. [32]	USA (50 States and District of Columbia)	Region	Statewide mask mandates	Time to mask mandate adoption	Cumulative incidence rate ratios (at 14 day intervals)	States with mask mandates made at three to six months after CDC recommendation had a 1.61 times higher rate than those who implemented within 1 month (adjusted rate ratio=1.61, 95% CI: 1.23–2.10). States with mask mandates made after 6 months or with no mandate had 2.16 times higher rate than those who implemented within 1 month (adjusted rate ratio-2.16, 95% CI 1.64 – 2.88)
Joo et al. [37]	USA (10 states)	Region	Statewide mask mandates	Pre-post mandate comparison	Hospitalization growth rate	For age 18–39, weekly hospitalization rates declined by 2.2% (95% CI - 2.1, 6.4) within 3 weeks of implementation, and declined by 5.6% (95% CI 0.9, 10.4) ≥3 weeks after implementation For age 40–64, weekly hospitalization rates declined by 2.9% (95% CI 0.3, 5.5) within 3 weeks of implementation, and declined by 5.6% (95% CI 1.0, 10.2) ≥3 weeks after implementation For age ≥65, there was no statistically significant decline in hospitalization rates post implementation
Dasgupta et al. [34]	USA (all counties)	Region	Statewide mask mandates	Pre-post mandate comparison	Incidence	The overall probability of a county becoming a rapid riser (CDC definition) was lower among counties in states with statewide mask mandates (aPR 0.57; 95% CI 0.51–0.63) Association was more pronounced in nonmetropolitan counties (aPR 0.33; 95% CI 0.24–0.44) compared to large metropolitan counties (aPR 0.68; 95% CI 0.59–0.77)
Guy et al. [35]	USA (all counties)	Region	Statewide mask mandates	Pre-post mandate comparison	Incidence and mortality	Mask mandates were associated with a 0.5 percentage point decrease in daily COVID-19 case rates 1–20 days after implementation, and decreases of 1.1, 1.5, 1.7, and 1.8, 21–40, 41–60, 61–80, and 81–100 days, respectively, after implementation Mask mandates were associated with a 0.7 percentage point decrease in daily COVID-19 death growth rates 1–20 days after implementation, and decreases of 1.0, 1.4, 1.6 and 1.9, 21–40, 41–60, 61–80, and 81–100 days after implementation
Poppe [36]	Colombia, Chile	Region	Country mask mandates	Pre-post mandate comparison	Incidence	Mask wearing in public spaces reduced confirmed cases as indicated by difference between pre- and post-intervention slope

aPR, adjusted prevalence ratio; aIRRR, adjusted ratio of incidence rate ratios.

### 3.2 Types of studies and outcomes

Studies used various study designs and analytic methods to assess associations between mask-wearing and COVID-19 outcomes. The majority of studies reported the influence of mask wearing policies on SARS-CoV-2 incidence; four studies reported incidence and mortality [20,24,30,35], and one study reported incidence, hospitalization and mortality [27]. Analytical approaches included univariate and multivariable analysis, synthetic control method, and interrupted time series analysis.

All studies reported a rapid and significant reduction in incidence associated with mask wearing policies. A study from Jena city, Germany, reported a 15–75% reduction in SARS-CoV-2 infections over 20 days after mandatory introduction [9]. A study from 16 US jurisdictions estimated 230,000–450,000 averted cases over a 7 week period [17]. Another study, from Arizona, USA, reported a 75% reduction in the 7-day moving average of daily cases over a 4 week period [24]. Similarly, large reductions in adverse clinical outcomes were reported: a study from Delaware, USA, reported an 88% reduction in hospitalizations and a 100% reduction in mortality associated with stay-at-home orders, public mask mandates and contact tracing [27].

Key findings are provided in Table 1.

### 3.3 Assessment of methodological quality

The methodological quality of the included studies was variable, and overall study quality was considered acceptable for contributing evidence to the broad question of protective efficacy of community mask wearing. The majority of studies carried out either no or limited adjustment for area-level confounders such as the use of other control measures in addition to the initiation of mask policies; the few studies that did attempt to perform statistical adjustment were limited in their ability to measure other infection control measures and confounders. Eleven studies defined an appropriate time interval between intervention (e.g. implementation of mask policy) and outcomes. Only three studies noted the limitation of cross-level bias, and that it is not possible to determine the effects of masking policies on individuals based on ecological data [18] (Table 2).

**Table 2:** Summary of Study Designs

Study	Design	Analysis	Defined time lag between intervention and outcome	Concurrent changes that may affect outcome	Documentation of other public health and social measures (PHSM)	Other limitations
Mitze et al. [9]	Synthetic control method	Robustness checks Cross-validation tests	Yes	Yes	40 PHSMs	Cross-level bias Limited confounder adjustment
Lan et al. [19]	Time series	Linear regression	Yes	Yes	No	Cross-level bias No confounder adjustment
Chernozhukov et al. [20]	Structural equation model	Multivariable linear regression	Yes	Yes	Policy variables: stay-at-home, school closures, closure of restaurants, closure of movie theaters, and closure of non-essential businesses Mobility variables: transit, grocery, retail, workplaces	Cross-level bias Limited confounder adjustment Variability in guidance by state
Chiang et al. [21]	National cumulative data	None	No	Yes	Singapore: stay at home policy	Cross-level bias No confounder adjustment Variability in Recommendations
Cheng et al. [22]	Cross-sectional	Descriptive statistics	No	Yes	No	Cross-level bias No confounder adjustment
Bo et al. [23]	Cross-sectional	Generalized linear mixed model	Yes*	Yes	Quarantine, physical distancing, traffic restrictions	Cross-level bias Limited confounder adjustment; effect of each PHSM unclear Countries as unit of observation Variability in mandates
Lyu and Wehby [17]	Event study	Difference-in-differences like comparison	Yes*	Yes	Physical distancing, closure of schools, businesses, restaurants, gyms, cinemas	Cross-level bias Variability in mandates
Gallaway et al. [24]	Event study	None	No	Yes	Physical distancing, closure of school, stay-at-home orders, business closures, enhanced sanitation practices, employee mask wearing, symptom screening for all businesses operating a physical location, limited capacity for public events	Cross-level bias No confounder adjustment Variability in mandates
Leffler et al. [25]	Cross-sectional	Multivariable linear regression	No	Yes	Travel restrictions, stay-at-home orders	Cross-level bias No confounder adjustment Countries as unit of observation Variability in mandates
Van Dyke et al. [26]	Event study	Segmented regression	No	Yes	None	Cross-level bias No confounder adjustment Variability in mandates
Kanu et al. [27]	Event study	None	No	Yes	Stay-at-home order	Cross-level bias No confounder adjustment Variability in mandates
Zhang et al. [28]	Event study	Linear regression	No	Yes	Physical distancing, stay-at-home orders	Cross-level bias No confounder adjustment Variability in mandates
Zhang and Warner [33]	Event study	Linear regression	Yes	Yes	Shut downs and re-openings	Cross-level bias Limited confounder adjustment Variability in mandates
Rader et al. [29]	Ecological case-control study	Multivariable logistic regression	Yes	Yes	Physical distancing	Survey bias Limited confounder adjustment Variability in mandates
Li et al. [30]	Event study	Interrupted time series with a comparative design	Yes	Yes	Stay at home order	Cross-level bias No confounder adjustment
Rebeiro et al. [31]	Event study	Multivariable and piecewise Poisson regressions	No	Yes	No	Cross-level bias Unclear confounder adjustment Variability in mandates
Krishnamachari et al. [32]	Event study	Negative binomial regression	No	Yes	Stay at home orders and school closures	Cross-level bias Limited confounder adjustment Variability in mandates
Joo et al. [37]	Event study	Weighted linear regression	Yes	Yes	Stay at home order and business closures	Cross-level bias Limited confounder adjustment Variability in mandates
Dasgupta et al. [34]	Event study	Poisson regression	No	Yes	Stay at home orders	Cross-level bias Limited confounder adjustment Variability in adherence to mandates; Statewide orders may not necessarily reflect locally enforced orders
Guy et al. [35]	Event study	Weighted least-squares regression	Yes	Yes	Restaurant and bar closures, stay at home orders, bans on gatherings.	Cross-level bias Limited confounder adjustment Variability in mandates
Poppe [36]	Event study	Interrupted time series	Yes	Yes	Stay at home order	Cross-level bias No confounder adjustment Variability in mandates

* Sensitivity analysis.

The majority of studies provided no information about ascertainment of exposure (rate of mask wearing and level of compliance) or the population affected by the policies (where masks should be worn and by whom). This prevented any conclusions to be drawn about the effectiveness of different policies.

Details of the quality assessment for the individual studies are provided in Table 3.

**Table 3:** Risk of Bias of ecological studies

Study	Selection			Comparability	Outcome			
	Exposure group representative^a^	Ascertainment of exposure^b^	Population exposed^c^	Comparable groups^d^	Controls for confounders^e^	Assessment of outcome^f^	Appropriate time lag^g^	Statistical test^h^
Mitze et al. [9]	*	–	*	*	**	*	**	*
Lan et al. [19]	*	–	*	–	–	*	*	*
Chernozhukov et al. [20]	*	–	–	*	**	*	*	*
Chiang et al. [21]	*	–	–	–	–	*	–	–
Cheng et al. [22]	*	**	–	*	–	–	–	–
Bo et al. [23]	*	–	–	–	**	*	**	*
Lyu and Wehby [17]	*	–	*	–	**	*	**	*
Gallaway et al. [24]	*	–	–	–	–	*	–	–
Leffler et al. [25]	*	–	–	–	*	*	–	*
Van Dyke et al. [26]	*	–	–	–	–	*	–	*
Kanu et al. [27]	*	–	–	–	–	*	–	–
Zhang et al. [28]	*	–	–	–	–	*	–	–
Zhang and Warner [33]	*	–	–	*	*	*	**	–
Rader et al. [29]	*	**	*	*	**	*	**	*
Li et al. [30]	*	–	–	*	–	*	**	*
Rebeiro et al. [31]	*	–	–	–	*	–	*	*
Krishnamachari et al. [32]	*	–	–	–	–	*	–	*
Joo et al. [37]	*	–	*	*	*	*	**	*
Dasgupta et al. [34]	*	–	–	*	**	*	–	*
Guy et al. [35]	*	–	*	*	**	*	*	*
Poppe [36]	*	–	–	*	–	*	*	*

*Satisfactory.

** Good.

a If studies chose a sample which were truly or somewhat representative of the average in the target population, we assigned 1 star.

b If rate of mask wearing was assessed within the population, we assigned 1 star. If this included a measure of level of compliance, we assigned 2 stars.

c If details about where masks should be worn and by whom, we assigned 1 star.

d Where a comparison was made, the comparison group was appropriate (ie similar risk of outcome) or statistical adjustments were made, we assigned 1 star.

e If other policy-level factors were controlled for (such as physical distancing, stay at home order, closure of public venues, restriction of gatherings), we assigned 1 star. If community level factors were controlled for (such as community prevalence and population size) we assigned 2 stars.

f If study used case data corresponding to the target population, we applied 1 star.

g If an appropriate lag time was incorporated to account for timing of effects of mask introduction and assessment outcome, we assigned 1 star. If a sensitivity analysis was conducted using a range of time lags, we assigned 2 stars.

h If the statistical tests used to analyze the data was clearly described and appropriate, and the measurement of the association was presented with confidence intervals, we assigned 1 star.

## 4 Discussion

Masks have long been a key intervention for infection prevention and control. For SARS-CoV-2, WHO has consistently given advice on the use of masks for the general public. Guidance has evolved from advising mask wearing for people who were unwell and their care givers in January 2020 [38], to guidance in April 2020 establishing policies of the use of masks by decisions makers, such as the use by the general public when individuals cannot physically distance [39] with additional guidance provided on specific settings in which to wear masks, and information on mask compositions in June[40] and December 2020 [1].

A limited number of systematic reviews have summarized the effectiveness of community mask wearing to protect against transmission of respiratory diseases at the individual level [2,8,11,41]. A systematic review published in June 2020 concluded that face mask use could result in a large reduction in risk of infection but did not include any studies of community mask wearing for the prevention of COVID-19 [41]. A second systematic review, updated to April 2021, identified one randomized trial and 3 observational studies assessing community mask wearing for the prevention of COVID-19, and rated the strength of the evidence as low for a small reduction in risk for infection with any mask use [8].

This systematic review identified 21 studies that used ecological data to assess the effectiveness of mask wearing to protect against adverse health outcomes related to SARS-CoV-2 infections in community settings. All studies reported a protective benefit in terms of either reduced incidence, mortality, hospitalization, or a combination of these outcomes. This review summarized the literature up to March 2021, since which time a number of additional studies have reported a protective benefit impact of community mask wearing by the general public in community settings using ecological data [42], [43], [44], [45], [46]. While the results of these individual studies support the findings of this review, the large amount of research being done to assess community mask wearing warrants future systematic assessment.

Studies of mask usage can try to measure two different effects: reduction of general population transmission or specific reduction of risk to an individual. The ecological fallacy is less of a concern in the former because many of these ecological studies focus on estimating population level effects regardless of individual risk. As such, the use of population level data is a feature as much as it is a limitation.

WHO Guidelines Development Groups evaluate all forms of relevant available evidence. While ecological studies have been cited as evidence to advocate for the adoption of universal masking policies [47], there are a number of important limitations for making causal inferences, in particular the ecological fallacy, or cross-level bias (Table 4) [16]. The GRADE framework, which is widely used to support guideline development [48], is not well-suited to assess such studies. Due to their observational design and inherent limitations, applying GRADE to such studies would result in very low strength of evidence assessments. An important limitation of the evidence base reviewed here is that in all but two studies [22,29], mask policies were assessed as the exposure variable, without providing information about compliance to these policies. Moreover, policies requiring people to wear masks in the community are variable in terms of where and when and in whom mask wearing is required.

**Table 4:** Challenges in interpreting findings from ecological mask studies

**Diagnostic capabilities**
Accounting for changes in SARS-CoV-2 infection diagnostic capacity and types of diagnostic tools used is critical for interpreting changes in incidence associated with policy implementation [53]. Testing capacity was only assessed by one study included in this review [17]. More affluent regions may have a greater ability to detect COVID-19 cases than less affluent regions, including among asymptomatic individuals, and the practice of face mask wearing, including availability and quality, may also be related to area-level affluence (meaning that both exposure and outcome are influenced by affluence).
**Concurrent preventive interventions**
Policies mandating community mask wearing are accompanied by policies promoting other nonpharmaceutical interventions known to prevent SARS-CoV-2 transmission – notably physical distancing, schools and workplace closures, and hand hygiene. Seventeen studies considered one or more nonpharmaceutical interventions in addition to mask wearing, with statistical adjustments made in nine of these studies[9,17,20,23,25,29,31,33,37]; the remainder did not consider the influence of other nonpharmaceutical interventions. It is not possible to reliably estimate the benefit of face-mask policies over and above the benefits that are brought about by the other measures. Furthermore, the contribution of vaccines will need to be accounted for as vaccination programmes reach scale.
**Mask policy vs mask wearing**
Mask quality varies by type of mask fabric [54,55], and when and how they are worn [56]. Only 2 studies reported mask type [19,21] and two considered adherence to mask policies [22,29]. None of the studies evaluated individual-level adherence to and appropriate wearing of masks. The ability to categorize mask policies is relatively crude (i.e. requiring people to wear masks in the community could encompass a lot of variability in terms of where and when and in whom masks are required)
**Differences in virus transmission**
None of these studies accounted for the potential contribution of superspreading events [57] or the emergence of new SARS-CoV-2 variants of concern that have higher transmissibility [58] on incidence trends. While most studies were completed prior to the emergence of variants of concern, and it is uncertain that the contribution of such variants on incidence trends could be assessed through ecological analyses.
**Timing from policy to outcome**
There is a time lag between policy implementation and possible impact on incidence, followed by hospitalization, and then mortality. Interpreting studies on the effect of mask and other policies requires appropriate consideration of timing. There is also variability in the requirements of mask policies, including differences in exemptions for certain age groups, places of worship, and other specific settings [59].

Few studies assessed the possible influence of concurrent implementation of other preventive measures such as hand hygiene, physical distancing, working from home policies, closures of businesses, and policies limiting gatherings. Where attempts were made to control for such confounders this was based on the existence of policies supporting such measures, with little information about compliance to these policies. Considering these limitations, it is challenging to disentangle the effectiveness of a single policy and draw conclusions about the superiority of one policy compared to another [49]. Ecological studies are also unable to account for rapid changes that impact transmission dynamics, such as the appearance of new variants of concern or the phased introduction of vaccines with unknown effectiveness at scale.

To address these limitations, future research should consider approaches to improving the reliability of ecological data to inform policy and practice. The time interval between changes in mask policies/masking rates and assessment of outcomes is another important limitation. Trends may have already been observed at the time the policies were implemented and some findings may have been sensitive to the time periods selected for analysis. Future studies should account for the time element, for example by pre-defining the time periods assessed using plausible assumptions about the expected time that effects of mask policies would be expected, account for trends in infection rates when the mask policies are implemented, and perform sensitivity analyses on the periods selected for analysis to determine robustness of findings to assumptions regarding the temporal relationship. Finally, the existence of a policy alone provides no information about levels of compliance to the intervention, and future studies are encouraged to include an assessment of levels of compliance.

It has been proposed that cross-level bias can be reduced by supplementing ecological with individual-level information [50]; individual-level studies on masking generally support an association between mask use and decreased risk of infection in those wearing masks [8]. Certain study designs should be considered to improve the validity of assessing the effect of mask policies on COVID-19 outcomes. Longitudinal studies with individual-level data are an appropriate study design, as has been suggested for investigating the relationship between air pollution and increased risk of COVID-19 infection or adverse clinical outcomes [52]. Case-control studies could provide important information about risk estimates at an individual level, while controlling for the other relevant risk factors at both regional- and individual levels.

Notwithstanding these limitations, where direct evidence based on individual-level data exist, the results of ecological studies can be considered to provide low certainty evidence about the protective effect of mask wearing at the community level. In conclusion, the studies summarized by this review suggest that mask policies may reduce the population-level burden of SARS-CoV-2. The appropriate and safe use of suitable masks as well as proper storage and cleaning or disposal of masks are essential to make them as effective as possible; and that the use of masks be supplemented by a comprehensive set of measures.

## Date sharing statement

All data are available in the original published articles included in this review, and are available upon request.

## Funding

There was no funding source for this study. The corresponding author had full access to all the data in the study and had final responsibility for the decision to submit for publication.

## Declaration of Competing Interest

All authors have nothing to declare.

## Appendix Supplementary materials

Image, application 1
