# Supplementary material for: Mask use in community settings in the context of COVID-19: A systematic review of ecological data
Source: eClinicalMedicine. 2021 Jul 19;38:101024. doi: 10.1016/j.eclinm.2021.101024 (PMC8287197; doi:10.1016/j.eclinm.2021.101024)
Supplement: Supplementary file 1 [file mmc1.pdf]

Supplementary Appendix

Mask use in community settings in the context of COVID-19: systematic review of ecological data

1. Systematic review protocol

1. INTRODUCTION

The wearing of medical and non-medical masks by the general public in community settings is one intervention that is important for the reduction of SARS-COV-2. This systematic review aims to summarize the study designs, outcomes, and key quality indicators of using ecological data to evaluate the association between mask wearing and COVID-19 outcomes.

2. METHODS

2.1. Types of studies

- Studies that reported on the association between mask wearing and incidence, disease severity, and mortality at the ecological level.
- Studies that evaluate the effects of a masking policy on the population and do not assess both exposure and health outcomes at the individual level are considered ecological studies for this review.
- Pre-prints were excluded

2.2. Types of participants

- Adults, adolescents and children in community settings.
    - Outcomes will be disaggregated by age where appropriate

2.3. Types of outcomes

Incidence, disease severity, and mortality

2.4. Databases

The WHO COVID-19 database will be searched from 5 January 2020) to 05 March 2021:
https://search.bvsalud.org/global-literature-on-novel-coronavirus-2019-ncov/

This database is a compilation of over 40 searches conducted in resources such as PubMed, Embase, CINAHL, GIM. A full list of resources is listed here (https://www.who.int/docs/default-source/coronaviruse/who-covid-19-database/who-covid-19_sources_searchstrategy_20210105.pdf?sfvrsn=480292c0_9

2.5. Search strategy

A search strategy was developed combining the following terms: personal protection, face masks, face coverings, and face shields.

The following search strategy, developed by an information specialist, will be used:

https://search.bvsalud.org/global-literature-on-novel-coronavirus-2019-ncov/?output=site&lang=en&from=0&sort=&format=summary&count=20&fb=&page=1&skfp=&index=tw&q=%28%28%22personal+protection%22+OR+PPE+%29+AND++%28face+OR+facial%29%29+OR+mask*+OR++facemask*+OR+%22face+covering*%22+OR+n95+OR+masking++OR+%28%28face+or+facial%29+AND+%28covering*+OR+shield*%29+%29+OR+%22face+piece*%22&search_form_submit=

2.6. Restrictions
No date, language, or geographical exclusions will be applied.

3.0. Risk of bias
Risk of bias will be assessed using an adapted version of the Newcastle Ottawa scale to evaluate eight key domains for evaluating ecological studies:

- Representativeness of exposure group
- Ascertainment of exposure
- Population exposed
- Comparability of groups
- Adjustment for confounders
- Outcome assessment
- Appropriateness of time lag between mask intervention and outcome assessment
- Statistical methodology.

4.0. Quantitative synthesis
No quantitative synthesis will be conducted.

Contact
Nathan Ford, WHO, Geneva
Email: fordn@who.int
